# Needles of *Pinus halepensis* as Biomonitors of Bioaerosol Emissions

**DOI:** 10.1371/journal.pone.0112182

**Published:** 2014-11-07

**Authors:** Amandine Galès, Eric Latrille, Nathalie Wéry, Jean-Philippe Steyer, Jean-Jacques Godon

**Affiliations:** INRA, UR0050, Laboratoire de Biotechnologie de l'Environnement, Narbonne, France; NERC Centre for Ecology & Hydrology, United Kingdom

## Abstract

We propose using the surface of pine trees needles to biomonitor the bioaerosol emissions at a composting plant. Measurements were based on 16S rRNA gene copy numbers of *Saccharopolyspora rectivirgula*, a bioindicator of composting plant emissions. A sampling plan was established based on 29 samples around the emission source. The abundance of 16S rRNA gene copies of *S. rectivirgula* per gram of *Pinus halepensis* needles varied from 10^4^ to 10^2^ as a function of the distance. The signal reached the background level at distances around the composting plant ranging from 2 km to more than 5.4 km, depending on the local topography and average wind directions. From these values, the impacted area around the source of bioaerosols was mapped.

## Introduction

Total leaf surface on earth amounts to 1,000,000,000 km2 and thus provides one of the planet's largest surface areas [1] which also harbours one of the biggest microbial community with 10^8^ cell cm^−2^ [2]. The very diverse range of phyllosphere microbes includes both plant and human pathogens and methanotrophs. Phyllosphere bacteria include true ‘residents’ as well as ‘transient colonizers’. A transient microbial population can be the sign of environmental change in the surrounding air: for example, a momentary microbial emission [3]. For all such microbes, their dispersal, immigration and invasion occur in the main through the air [4], [5]. The source of these microbes released into the atmosphere include bioaerosols from plant foliage, soil dust, sea-sprays along with other environmental sources [5], [6]. Moreover, a wide range of anthropic activities is also the origin of bioaerosols when processes involve the disturbance of biological matter. Composting is reliant on the presence of various microorganisms which may become airborne; thus, the dispersal of bioaerosols from the site must be examined. The area impacted by bioaerosols offers benefits on prokaryotic biogeography, on ecological features and on epidemiology of human, animal and plant pathogens to research [7]. Airborne bacteria and moulds are major contributors in humans allergic or respiratory diseases [8], [9]. Air sampling on site for assessing the area impacted by composting bioaerosols is a current method. However, even if the determination of specific indicators enhances monitoring performance [10], parameters which influence the instantaneous measurements involved make it difficult to reproduce and interpret a study[11]. In fact, meteorological conditions and sampling methods greatly influence any analysis in terms of distance travelled and dispersal.

In order to record the spatial distribution of bioaerosol emissions at a composting plant, we employed a biomonitoring approach based on vegetation of pine trees (*Pinus halepensis*). We used the interaction between airborne microbes and the phyllosphere to measure the response of ecosystems to changes in their environment affecting atmospheric air [12], [13]. The development of tools and techniques to improve biomonitoring is a prerequisite for a better integration of the assessment of exposure to microbial emissions and epidemiological research. Vegetation biomonitoring has already been used for examining local source-receptor relationships in the way of determining the magnitude and shape of the PAH spatial distribution [14].

In the present work, we took advantage of the ability of leaf surfaces to accumulate microbes from the air [5]. Our aim, which today representing a real challenge, was to map the impacted area around a located source of bioaerosols.

## Material and Methods

### Ethics statement

Samples were not collected in protected areas. The areas sampled belonged to a city where no specific permission was required. Field studies did not involve endangered or protected species.

### Field collection

The source of bioaerosols, located in the south of France, was a composting plant surrounding by a pine forest (*Pinus halepensis*) without any known large source of bacterial emissions. The sampling sites were chosen based on a sampling plan centered on the composting plant at distances from 100 m to 6000 m of the platform center. 29 samples of pine needles were collected.

Three control samples of *P. halepensis* needles were collected at 150 km (two locations) and 300 km distance to provide background median values. The control chosen areas were a long way from any industrial composting facility and the surroundings of the control locations were very different one from each other: urban area, scrubland and pine forest.

For each measuring campaign around the composting site, the needles required to draw a spatial model of bacterial abundance were sampled in one period. The two campaigns (September and November 2012) took place during less than 24 hours without any rainfall event. The collection made in autumn included needles aged approximately 6 months to 3 years, based on the fact that conifer has a single annual growth period in the spring.

Needles were collected from a minimum of two branches (on branch ends: about 20 cm) on each tree at 1.5–2 m height as the bacterial communities leaf surface is likely to vary with canopy height [15]. The branches chosen were from opposite sides of the tree, randomly, to limit any bias in the collection process. For each tree, branches were wrapped in the same plastic bag and stored at ambient temperature during the collection period. For each sample, needles were all detached and thoroughly mixed to ensure that needle age did not affect the analyses. Then, 15 g (corresponding to 30% of the sample) was taken from the mix and washed to harvest phyllosphere bacteria.

Pinus needles were washed inside containers with a 1∶50 diluted sterile wash solution (1 M Tris–HCL, 500 mM ethylenediamine tetraacetic acid, and 1.2% Triton diluted in sterile water) [16] and the container was shaken for 5 min prior to incubation at room temperature for 2 hours. The wash solutions were then centrifuged at 2,000×g for 10 min. The supernatant was discarded and the DNA was extracted from the pellets.

### DNA extraction and 16S rRNA gene quantification

The liquid phase was centrifuged and DNA extraction was performed on the pellets as previously described [17]. 16S rRNA gene abundance of total bacteria and *S. rectivirgula* were measured by qPCR and expressed as log_10_ [copy per gram needle] as previously described [17], [18].

### Spatial modeling

Based on the 29 samples, the spatial abundance of *S. rectivirgula* and total bacteria 16S rRNA gene copies on *P. halepensis* needles was mapped using a thin plate spline regression function (Tps function) of the “Fields package” [19] of R software [20].

## Results and Discussion

**Figure 1:**
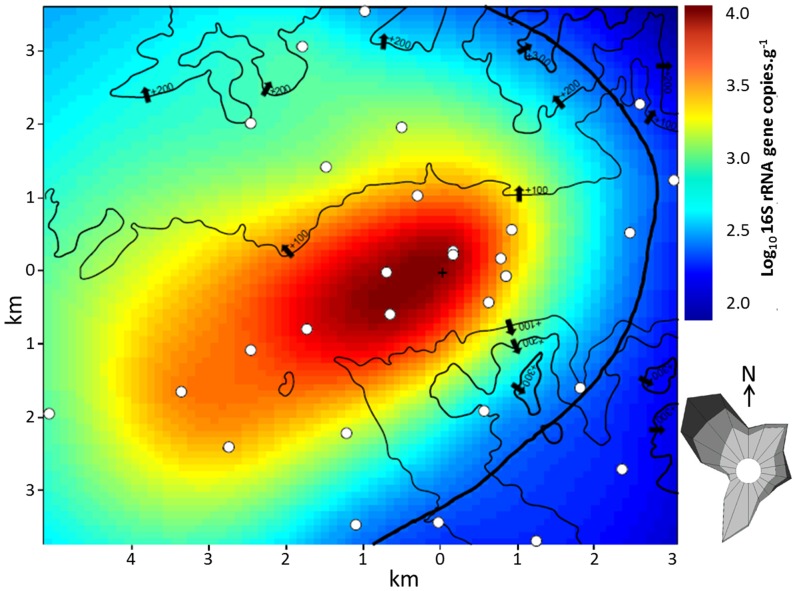
Predicted *S. rectivirgula* concentration in 16S rRNA gene copies.g^−1^ of *Pinus halepensis* needles. White spots correspond to the location of sampled trees. Narrow and thick black lines correspond to altitude and background level contour lines, respectively. The cross corresponds to the location of the composting plant. The wind map shows the frequency of no wind (0 to1.5 m.s^−1^), light wind (1.5 to 4.5 m.s^−1^), moderate wind (4.5 to 8 m.s^−1^) and strong wind (up to 8 m.s^−1^) in, respectively, white, light grey, medium grey and dark grey (Meteo France, http://www.meteofrance.com/).

**Figure 2:**
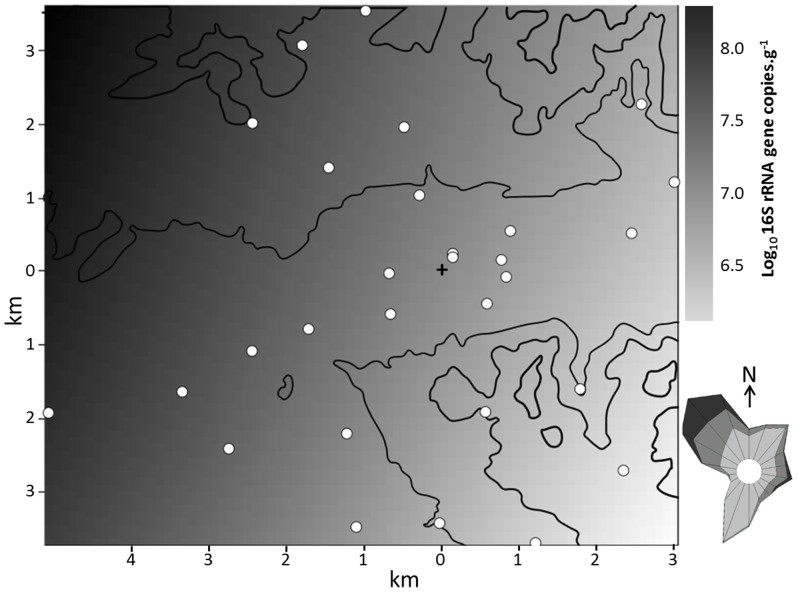
Predicted total bacteria concentration in 16S rRNA gene copies.g^−1^ of *Pinus halepensis* needles. White spots correspond to the location of sampled trees. Narrow black lines correspond to altitude. The cross corresponds to the location of the composting plant. The wind map shows the frequency of no wind (0 to1.5 m.s^−1^), light wind (1.5 to 4.5 m.s^−1^), moderate wind (4.5 to 8 m.s^−1^) and strong wind (up to 8 m.s^−1^) in, respectively, white, light grey, medium grey and dark grey (Meteo France, http://www.meteofrance.com/).

In order to determine the degree of microbial accumulation, we took advantage of the waxy surface of *Pinus halepensis* needles that enables them to accumulate up to 3 years, focusing on and *S. rectivirgula* which was considered as an indicator of compost bioaerosols [17], [18]. From 29 samples collected around the composting plant, the maximum abundance (4.40±0.02) of *S. rectivirgula* per gram of *Pinus halepensis* needles was detected closest to the source. The signal fell to the background level (2.00±0.03) at distances ranging from 2 km to more than 5.4 km from the composting plant (Table 1). Background was defined from needles collected more than 150 km from the site (see Material and Methods). The dispersion map of *S. rectivirgula* confirms an inverse correlation between the distance from the emission source and the concentration of the microbiological indicator (Figure 1). However, the dispersion map of the bioindicator does not form a circle around the source but stretches along the valley on account of the topography and the wind (Figure 1). In contrast to the variability in *S. rectivirgula* measurements, the 16S rRNA gene concentration targeting total bacteria appeared relatively stable across samples. The concentration varied from 5.5 to 8.3 (Table 1) with an average of 7.14±0.01. The total bacteria dispersion map shows no correlation with distance to the composting plant and shows a weak and unexplained North-West South-East gradient (Figure 2).

**Table 1:** Concentration of 16S rRNA gene copies of *S. rectivirgula* and total bacteria on *Pinus halepensis* needles around a composting plan.

West/East distance (km)	North/South distance (km)	Distance from plant (km)	*S.rec* 1	Bact 2
0.16-E	0.22-N	0.28	4.4	7.9
0.16-E	0.26-N	0.31	4.0	6.2
0.69-W	0.04-S	0.69	3.6	6.9
0.62-E	0.43-S	0.75	3.0	6.9
0.79-E	0.18-N	0.80	3.0	6.3
0.84-E	0.06-S	0.84	2.9	6.5
0.66-W	0.57-S	0.87	4.1	7.9
0.91-E	0.56-N	1.07	2.6	8.3
0.29-W	1.04-N	1.08	2.8	7.7
1.74-W	0.78-S	1.90	2.6	7.9
0.58-E	1.90-S	1.98	2.0	6.2
0.49-W	1.97-N	2.03	2.9	8.0
1.48-W	1.42-N	2.05	2.5	7.7
1.81-E	1.58-S	2.40	2.0	7.4
2.47-E	0.53-N	2.52	2.0	6.1
1.22-W	2.22-S	2.53	2.8	7.7
2.46-W	1.08-S	2.68	3.0	6.8
2.46-W	2.02-N	3.18	2.4	6.8
3.03-E	1.24-N	3.26	2.0	7.3
0.03-W	3.42-S	3.42	1.9	5.5
2.59-E	2.29-N	3.45	1.9	7.0
1.79-W	3.07-N	3.56	2.8	7.6
2.36-E	2.69-S	3.58	1.9	5.6
1.10-W	3.45-S	3.62	2.0	8.0
2.75-W	2.41-S	3.65	3.2	8.0
0.98-W	3.56-N	3.69	2.0	6.8
3.36-W	1.64-S	3.73	3.4	8.2
1.24-E	3.68-S	3.88	2.0	6.3
5.07-W	1.93-S	5.41	2.2	7.5

1 , *S. rectivirgula*;

2 , total bacteria expressed as log [copy per gram needle].

**Figure 3:**
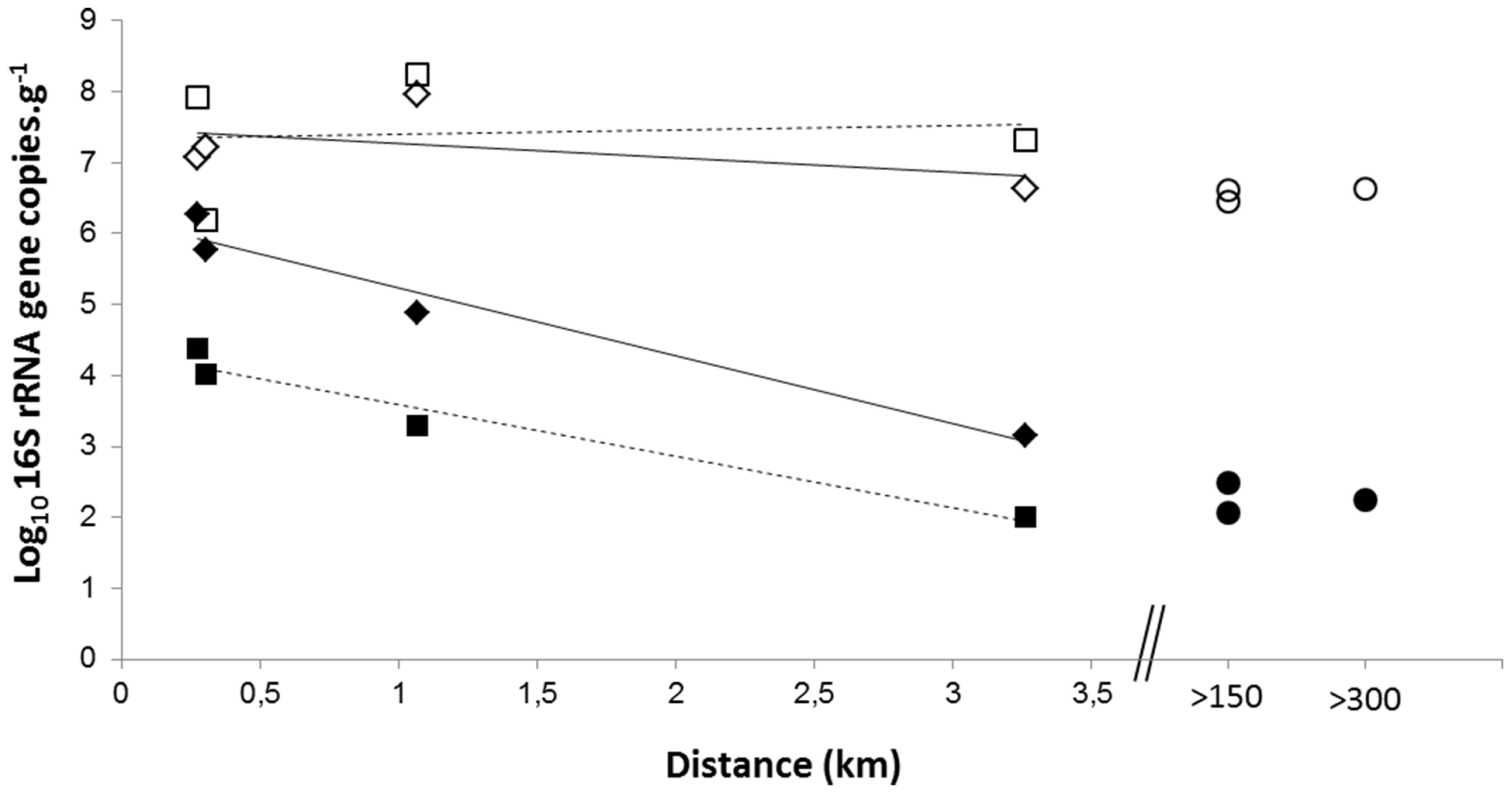
Spatial dynamics for two different time periods. Diamond and full line correspond to samples collected in September whereas the squares and dashed lines correspond to samples collected at the same location in November. Circles correspond to background samples. White and black symbol corresponds to total bacteria and *S. rectivirgula* respectively.

Successional changes occur in the microbial communities of the phyllosphere over a season [15], [16]. To test the robustness of the approach, we sought to determine the behavior of accumulation over time. To this end, we compared four samples along a transect collected in September and November. No significant difference was observed at total bacterial levels between samples (Figure 3). In contrast, the number of *S. rectivirgula* decreased in all second samples but remained inversely correlated to distance from the emission source (Figure 3). Importantly, this concentration of *S. rectivirgula* changed over time but did not modify the map of the impacted area around the source of bioaerosols. The difference between the two sampling periods may be explained by an important rainfall event which occurred between the two sampling dates. Thus, the number of *S. rectivirgula* depends on the topography, the distance from the emission source and meteorological parameters such as wind direction or rain, in contrast, the number of total bacteria does not seem to be affected by the same parameters. Despite its stability, the number of total bacteria shows only slight variations on a larger geographical scale, with origin remaining unknown. In non-impacted areas on needles, the the number of 16S rRNA gene copies of *S. rectivirgula* was around 10^2^ and total bacteria were around 10^7^ per gram whereas in air the number of 16S rRNA gene copies of *S. rectivirgula* is around 10^2^ [10] and total bacteria is around 10^5^ per cubic meter [21]. Thus, the phyllosphere only gives a partial and distorted picture of the airborne microbes.

Nowadays, standard bioaerosol samplers provide a point measurement under site- and time- specific conditions (plant emission level and meteorology). The assessment of exposure based on measurement of bioaerosols remains an unresolved challenge requiring many measurements and also a modelling component. In contrast, pine needles considered as passive air samplers allow the direct mapping of impacted areas around a bioaerosol source that take into account emission levels as well as geographic and meteorological parameters without the need for measuring these parameters. Such accumulative measurements encompassed dry deposition varying over time as well as several rainfall events, thus reducing impacte from one-off meteorological events. Despite these advantages, pine needles do not provide data on the flux or concentration of emitted airborne microbes which are provided, at least for a defined period of time, by standard bioaerosol samplers. Bioaerosol impact maps, independent of any other measurements, should be very useful for validating models of bioaerosol dispersal in the environment. Moreover, this raises the question of the particle forms emitted by compost (aggregate, rafted particle or single cell) that greatly influence their atmospheric residence time, the distances travelled and their impact on health. Such data on an impacted area combined with a dispersal model should make it possible to determine the range of particle size of the composting bioaerosols.

To sum up, this study demonstrated that needle surfaces retained the imprint of airborne microbial pollution, underlining the ability of airborne microbes to adhere to leaf surfaces. These findings provide the basis for a new strategy in environmental biomonitoring.

Using cumulative data, a wide range of ecological concepts can now be considered, these include the immigration of microbes [22], monitoring of dispersal of plant pathogens or the presence of human pathogens and exposure assessment in epidemiology studies.
